# The potential role of precision medicine to alleviate racial disparities in prostate, bladder and renal urological cancer care

**DOI:** 10.1002/bco2.323

**Published:** 2024-02-08

**Authors:** Kunal K. Sindhu, Zachary Dovey, Marcher Thompson, Anthony D. Nehlsen, Karin A. Skalina, Beata Malachowska, Shaakir Hasan, Chandan Guha, Justin Tang, Lucas Resende Salgado

**Affiliations:** ^1^ Department of Radiation Oncology Icahn School of Medicine at Mount Sinai New York NY USA; ^2^ Department of Urology Icahn School of Medicine at Mount Sinai New York NY USA; ^3^ Department of Radiation Oncology AIS Cancer Center/Adventist Health Bakersfield CA USA; ^4^ Department of Radiation Oncology Montefiore Medical Center/Albert Einstein College of Medicine Bronx NY USA

**Keywords:** genito‐urinary cancers, precision medicine, racial disparity, social determinants of health, trial recruitment, tumour biology

## Abstract

**Background:**

Racial disparities in oncological outcomes resulting from differences in social determinants of health (SDOH) and tumour biology are well described in prostate cancer (PCa) but similar inequities exist in bladder (BCa) and renal cancers (RCCs). Precision medicine (PM) aims to provide personalized treatment based on individual patient characteristics and has the potential to reduce these inequities in GU cancers.

**Objective:**

This article aims to review the current evidence outlining racial disparities in GU cancers and explore studies demonstrating improved oncological outcomes when PM is applied to racially diverse patient populations.

**Evidence acquisition:**

Evidence was obtained from Pubmed and Web of Science using keywords prostate, bladder and renal cancer, racial disparity and precision medicine. Because limited studies were found, preferred reporting items for systematic reviews and meta‐analyses (PRISMA) guidelines were not applied but rather related articles were studied to explore existing debates, identify the current status and speculate on future applications.

**Results:**

Evidence suggests addressing SDOH for PCa can reverse racial inequities in oncological outcomes but differences in incidence remain. Similar disparities in BCa and RCC are seen, and it would be reasonable to suggest achieving parity in SDOH for all races would do the same. Research applying a PM approach to different ethnicities is lacking although in African Americans (AAs) with metastatic castrate‐resistant prostate cancer (mCRPCa) better outcomes have been shown with androgen receptor inhibitors, radium‐223 and sipuleucel. Exploiting the abscopal effect with targeted radiation therapy (RT) and immunotherapy has promise but requires further study, as does defining actionable mutations in specific patient groups to tailor treatments as appropriate.

**Conclusion:**

For all GU cancers, the historical underrepresentation of ethnic minorities in clinical trials still exists and there is an urgent need for recruitment strategies to address this. PM is a promising development with the potential to reduce inequities in GU cancers, however, both improved understanding of race‐specific tumour biology, and enhanced recruitment of minority populations into clinical trials are required. Without this, the benefits of PM will be limited.

## 1 INTRODUCTION

According to CDC statistics, in the United States, over 300 000 urological cancers have been diagnosed each year since 2013, and 1 in 3 cancers in all men are urological in origin. Prostate cancer (PCa) was the most common in all racial groups as well as having the highest overall incidence in non‐Hispanic Black men, whereas bladder (BCa) and renal cancers (RCC) are the second and third most common cancers, respectively, in Asian, Pacific Islander and non‐Hispanic White (NHW) men.^1^ With these figures in mind, there are concerning and significant ethnic and racial disparities in quality of care and treatment outcomes for all GU cancers. Several factors contribute to these inequities, including unequal access to care, poor clinical trial recruitment, socioeconomic and cultural factors in the generation of treatment plans and ethnic differences in tumour biology. In recent years, precision medicine (PM) has emerged as a promising approach to cancer care, aiming to provide individualized treatment to patients based on the genetic and molecular characteristics of their tumours. This paper will explore the potential role of PM in addressing racial disparities in GU cancers and discuss its implications for improving patient outcomes.

## 2 RACIAL DISPARITIES IN GENITOURINARY CANCERS

**FIGURE 1:**
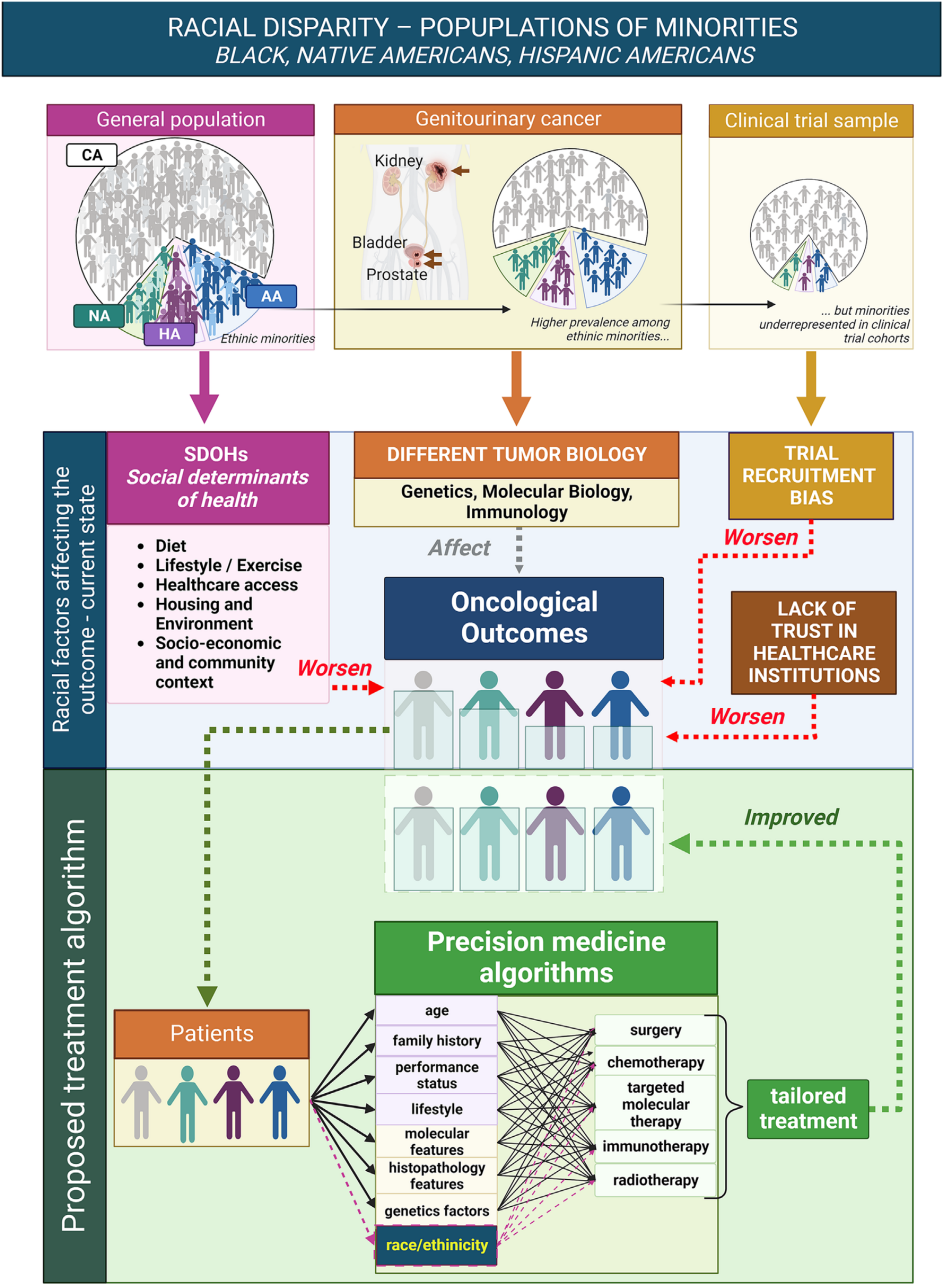
Illustrating factors influencing oncological outcomes in ethnic minorities, and the potential benefit of a precision medicine approach (African American [AA], Hispanic American [HA], Native American [NA], social determinants of health [SDOH]).

(See Figure 1 illustrating factors influencing oncological outcomes in ethnic minorities, and the potential benefit of a PM approach).

### 2.1 Prostate cancer

#### 2.1.1 Epidemiology

Nearly 1 in 5 men in America will be diagnosed with PCa in their lifetime. Black men are 60% more likely than White men to be diagnosed with PCa and are 200% more likely than White men to die of the disease. In the United States, the lifetime risk for African American (AA) men of dying from PCa is twice that of NHW men. Hispanic men have increased odds of high‐risk localized PCa than White men but are less likely to receive treatment. By contrast, Hispanic Black men are less likely to have high‐risk localized disease than non‐Hispanic Black men but are more likely to receive treatment.^2^ These racial disparities are believed to result from differences in social determinants of health (SDOH) (for example, lifestyle and diet, social and cultural networks, healthcare access issues and comorbidities) and tumour biology.

#### 2.1.2 Lifestyle factors

Diet, obesity and lifestyle are increasingly regarded as important factors in PCa incidence and treatment outcomes, although there is a paucity of research relating these factors to race. Epidemiological study shows men migrating to the West, with long‐term exposure to a Western diet (including fat, red meat, alcohol and dairy products), may increase the risk of PCa. However, studies focused on specific food items in western AA populations such as processed meats, poultry, fatty acids or plant‐based foods have been inconclusive.^3^ Obesity has been linked to PCa risk and disease progression^4^ and although AA men have a higher prevalence of obesity than Caucasian (CA) men, the difference is small.^5^ Nevertheless, a retrospective analysis of obesity as a predictor of adverse outcomes found it was linked to aggressive PCa in both AA and CA populations.^6^

#### 2.1.3 Healthcare access

Relatively recently, two landmark epidemiological studies found once socioeconomic issues were equalized, differences in treatment outcomes were eradicated. One examined 306 100 patients with localized or locally advanced PCa from Surveillance, Epidemiology and End Results (SEER), Veterans Affairs (VA) Health System, and four pooled National Cancer Institute Radiation Therapy Oncology Group phase 3 randomized controlled trial databases, over 55 482 (18.1%) of whom were AA men, and the other a cohort of 60 035 PCa patients from the VA Health System, 30.3% of whom were AA. Once treatment was standardized and healthcare access issues resolved, stage for stage PCa mortality in AA men was not significantly different from NHW men in both studies.^7^, ^8^ Another SEER study found that AA men with localized PCa were significantly less likely to receive definitive therapy compared with NHW men, regardless of tumour grade or risk group. Hispanic men with intermediate or high‐risk diseases were also less likely to receive definitive treatment.^9^ A subsequent metanalysis evaluating the impact of SDOH on PCa outcomes in Black and White patients studied over 1 million patients from 47 studies and confirmed a significant link between race, SDOH and PCa outcomes, and reiterated the importance of addressing SDOH to achieve equitable treatment outcomes.^10^

#### 2.1.4 Clinical trial recruitment

Patients from minority backgrounds are underrepresented in PCa clinical trials.^11^, ^12^ An analysis of enrollment in clinical trials leading to cancer drug approvals from 2008 to 2018 reported just 3.1% of enrolled patients were Black; worse, the proportion of enrolled Black patients had declined from 3.6% to 2.9% over the decade. Hispanic enrollment is also underrepresented, making up just 6.1% of the enrolled patient population over the examined decade. Several issues must be overcome to improve trial recruitment for minority populations including eligibility criteria ruling out minorities with certain comorbidities, institutional locations that may favour more affluent populations, insurance problems and travel costs. There is a paucity of research into developing recruitment strategies to overcome these problems although one recurring theme is the importance of trust between minority communities and research institutions, as well as identifying leading community advocates to facilitate communication and understanding.^3^

#### 2.1.5 Tumour biology

Although PCa genetic risk may be offset with a healthy lifestyle across all ethnic groups,^13^ there is also evidence Black, Hispanic American (HA) and White populations may have fundamental differences in PCa tumour biology that influence a ‘precision’ treatment approach. In one of the largest comparative studies on this topic, data from a cohort of 1152 men (596 AA and 556 NHW) undergoing surgery for localized PCa at a single centre by a single surgeon were examined retrospectively.^14^ On post‐surgical pathology specimens, AA men had significantly higher genomic scores for low‐risk Gleason grade groups (GGGs) (GGG 1 and 2), whereas NHW men had significantly higher genomic scores for high‐risk GGGs (GGG 4 and 5). Additionally, NHW men were found to have increased ETS‐related gene (ERG) and E26 transformation‐specific (ETS) expression, reduced serine peptidase inhibitor Kazal type 1 (SPINK1) expression and a predominance of basal‐type molecular genes. Conversely, AA men had increased expression of glutathione S‐transferase mu 3 (GSTM3), beta‐crystallin B2 (CRYBB2) and inflammatory genes (chemokine ligands 4 [CCL4], interleukin 33 [IL33], cluster of differentiation 3 [CD3], interferon gamma [IFNG], inducible T‐cell costimulator ligand [ICOSLG]) but lower mismatch repair gene expression (mutS homolog 6 [MSH6], mutS homolog 2 [MSH2]).

Examining molecular pathway activity for pathways implicated in PCa development and progression, NHW men have increased activity in DNA repair, fatty acid and glycolytic metabolism and WNT/beta‐catenin gene signalling, whereas AA men have higher activity in immune response, hypoxia, reactive oxygen and apoptosis‐related genes. Based on their increased expression of inflammatory genes, their findings advocate the use of immunotherapy in AA men, which supports the findings of a subanalysis of the Provenge Registry for the Observation, Collection, and Evaluation of Experience Data (PROCEED) trial demonstrating a survival advantage in AA men with metastatic castrate‐resistant prostate cancer (mCRPCa) treated with sipuleucel‐T.^14^ Other studies examining tumour mutations by race have shown Black men have higher mutation frequency in actionable genes (e.g. epidermal growth factor receptor [EGFR], v‐erb‐b2 avian erythroblastic leukemia viral oncogene homolog 2 [ERBB2], breast cancer gene 1/2 [BRCA1/2] and phosphatidylinositol‐4,5‐bisphosphate 3‐kinase [PIK3CA]) in primary and metastatic PCa and in androgen receptor and DNA repair genes in metastatic PCa compared with White and Asian men.^15^, ^16^ Using genomic analysis of tumour tissue to plan PM has promise, but once again incorporating genomic analysis into clinical trials has been highly Eurocentric. As research in genomic prognostic models expands, it is crucial that the data from which these algorithms are developed includes minority populations, or even better, prognostic models are developed that are themselves ethnic‐specific.

### 2.2 Bladder cancer

#### 2.2.1 Epidemiology

Black race has been found to be an independent predictor of delayed diagnoses and treatment for BCa and inferior quality of care.^17^, ^18^ A National Cancer Database (NCDB) study of BCa found AA patients have a higher proportion of muscle invasive bladder cancer (MIBC) and higher grade disease.^19^ A SEER database study showed Black patients were significantly less likely to undergo radical cystectomy (RC), which is strongly associated with overall survival, and another analysis from the NCDB reported that Black patients with MIBC undergoing RC were less likely to undergo pelvic lymph node dissections (PLNDs) and among those who underwent PLND, fewer lymph nodes were removed, both of which have been associated with improved outcomes.^17^, ^20^ Similarly AA race is independently associated with a higher risk of recurrence post RC and worse overall survival for metastatic disease.^21^, ^22^

A SEER study collecting data from the 1970s to 1990s found a disproportionately higher rate of delayed diagnosis and treatment among AAs for BCa, and despite a recognition of these disparities, more contemporary real‐world data from the last two decades has shown no improvement. In fact, the most recent NCDB analysis revealed, except for uninsured patients, Black race was the strongest independent predictor of BCa diagnosis at a later stage, and this discrepancy only widened as the stage advanced.^23^

#### 2.2.2 Tumour biology

An understanding of any differences in tumour biology is crucial to PM initiatives. Regardless of ethnicity, current PM bladder cancer research has focused on personalizing treatment based on P53 status, fibroblast growth factor (FGFR) expression and alterations, human epidermal growth factor receptor (HER) targeting, PIK3CA alterations, mutations in DNA damage response and repair genes and BCa molecular subtypes.^24^ However, any racial differences based on the above proposed categories for personalized therapies or the differences in higher grade disease in AA populations found in the NCDB study are poorly understood. Studies focused on identifying specific genomic subtypes of bladder cancer unique to AA patients have found, for example, EGF containing fibrulin‐like extracellular matrix protein 1 (EFEMP1), S100 calcium binding protein A16 (S100Ar16), and myeloid cell leukemia 1 (MCL1) may be overexpressed in AA patients.^25^ EFEMP1 overexpression is a plausible mechanism contributing to muscle invasion and elevated S100A16 protein has been shown to inhibit apoptosis via the alpha serine/threonine‐protein kinase/B‐cell lymphoma 2 (AKT/Bcl‐2) pathway in mitomycin C (MMC) resistant cell lines, whereas suppression of S100A16 may restore MMC sensitivity.^26^ MCL1 is an anti‐apoptotic protein of the B‐cell lymphoma 2 (BCL2) family and its downregulation is linked to tumour necrosis factor‐related apoptosis‐inducing ligand (TRAIL) sensitization such that therapeutic MCL1 inhibition may potentially enhance the effect of Bacillus Calmette‐Guerin (BCG) in non‐MIBC.^25^

The same study also found an aggressive genetic subtype, known as C3, is associated with a worse response to surgery and a better response to immune checkpoint inhibitors. Given that AA patients have reduced transcription levels of the genes associated with this aggressive C3 subtype, they may have better outcomes with surgical treatment as opposed to immune checkpoint inhibitors.^25^, ^27^

### 2.3 Renal cancer

#### 2.3.1 Epidemiology

RCC has an incidence of 2%–3% per year which is rising.^28^ Although RCC research in minority populations is generally lacking, AA, Native American (NA) and HA men have an increased risk of RCC,^29^, ^30^ and NAs and HAs are more likely to be diagnosed at a younger age. AA men have a higher incidence of localized disease at diagnosis although studies indicate AA men have increased mortality compared with age and stage‐matched controls.^31^ Another study came to the same conclusion in a cohort of patients from the International Marker Consortium for RCC database which showed AA patients who recur had an increased risk of cancer‐specific death and that the cause of this is likely multifactorial resulting from both SDOH and differences in tumour biology.^32^

#### 2.3.2 Tumour biology

RCC classification is closely linked to its tumour biology and as our understanding of RCC molecular biology has improved, molecular subtypes have been added, including the most recent WHO classification in 2022.^33^ As well as the histologically defined RCCs (clear cell, papillary, oncocytic and chromophobe, collecting duct and histological variants), there are now 11 categories of molecularly defined renal cancers, for example, TFE3‐rearranged RCC, TFEB‐altered RCC, ELOC (formerly TCEB1)‐mutated RCC, fumarate hydratase‐deficient RCC, succinate dehydrogenase‐deficient RCC, ALK‐rearranged RCC and three categories of SWI/SNF‐related matric associated actin‐dependent regulator of chromatic subfamily B member 1 (SMARCB1) (INI1)‐deficient RCC.

Similarly, in recent years, RCC driver mutations have been investigated, most notably, the loss of heterozygosity of the von Hippel–Lindau (VHL) suppressor gene. This has led to the development of therapeutic targets for the vascular endothelial growth factor (VEGF) pathway with drugs such as Pazopanib and Sunitinib, providing viable alternatives to the mammalian target of rapamycin (mTOR) targeting agents.^34^ Subsequently genomic studies have shown intrinsic differences in RCC between AA and White patients, focusing specifically on the nine most common mutations: *VHL*, protein polybromo‐1 human (*PBRM1*), SET domain containing 2 (*SETD2*), lysine‐specific demethylase 5C (*KDM5C*), phosphatase and tensin homolog (*PTEN*), BRCA1‐associated protein 1 (*BAP1*), *mTOR*, tumor protein p53 (*TP53*) and phosphoinositide 3‐kinase (*PI3KC*). Finding, for example, AAs have less frequent VHL mutations suggesting VEGF targeted therapy may be less efficacious.

On the basis of an additional classification of clear cell RCC into CC‐e.1, CC‐e.2 and CC‐e.3, where ‘e’ stands for ‘enriched’ on genomic analysis with clustering on a heat map,^35^ the same study found AAs have a higher frequency of CC‐e.3 subtype compared with EAs, less PBRM1 mutations in those AAs with CC‐e.1 and CC‐e.2 subtypes compared with EAs and higher frequency of BAP1 mutations in those AAs with CC‐e.3 subtype compared with EAs.^36^ By contrast, the frequencies of different molecular subtypes in HAs and NAs remain poorly investigated, despite, for example, HAs and NAs having a higher incidence of RCC along the US–Mexico border, as well as a higher prevalence of risk factors such as chronic renal disease and obesity.^30^ The use of PM is unlikely to be available for these populations until such time as their recruitment to clinical trials is improved.

## 3 PRECISION MEDICINE AND ITS POTENTIAL BENEFITS IN ALLEVIATING RACIAL DISPARITIES

(See Table 1 listing current GU cancer clinical trials focused on PM with illustration of their inclusion of racial diversification in recruitment or outcomes and Figure 1 illustrating factors influencing oncological outcomes in ethnic minorities, and the potential benefit of a PM approach).

**TABLE 1:** GU clinical trials focused on precision medicine in GU cancers.

Trial name	Trial location	Cancer type	Type	Primary outcome(s)	Planned # pts	Plan for racial equity in recruitment	Status	Projected completion date
Prostate cancer								
NCT05396872 Patient Decision‐making About Precision Oncology in Veterans With Advanced Prostate Cancer	University of California, San Francisco	Advanced prostate cancer	Interventional	To evaluate patient‐participants' decisional conflict about precision oncology and develop a Decision Support Intervention (DSI) to improve decision‐making	117	Secondary objectives include evaluation of patient‐participants', caregiver‐participants' and provider decisional needs, potential solutions, and potential disparities about precision oncology	Recruiting	7/31/27
NCT04706663. A Multi‐Center Natural History Study of Precision‐Based Genomics in Prostate Cancer	La Jolla, California	Histologically conformed prostate cancer	Observational	To assess the influence of specific gene mutations on disease course (germline and/or somatic variants in PIK3 and/or AKT, PALB2, BRIP1, RAD50, RAD51, RAD54, RB1, SPOP, Wnt/B‐catenin pathway, CDK12, and MMR genes: MLH1, MSH2, MSH6, PMS2, and EPCAM as well as subjects with tumour mutational burden‐high (TMB‐H) prostate cancer	2000	No	Recruiting	6/30/26
NCT03327675 An AI Platform Integrating Imaging Data and Models, Supporting Precision Care Through Prostate Cancer's Continuum	Fondazione del Piemonte per l'Oncologia, Turin, Italy	Histological confirmed PCa or suspicion of PCa (abnormal PSA values and/or positive DRE)	Observational (patient registry)	To develop vendor‐specific and vendor neutral AI models exploiting the prospective data that will be uploaded to the Prostate‐NET platform.	17 000	No	Recruiting	9/30/24
NCT04763317 Precision Medicine in the Prostate Cancer Care Pathway: an Evaluation of Integrating Germline Genetic Testing Into the Management of Men at Risk of/Living With Prostate Cancer	Institute of Cancer Research, United Kingdom Royal Marsden NHS Foundation Trust	Prostate cancer and those with a genetic predisposition to prostate cancer	Observational	To determine the prevalence of prostate cancer (PrCa) specific genetic variation in men with: (a) young onset PrCa; (b) metastatic PrCa; (c) men with PrCa and a family history of PrCa compared with controls.	2000	No	Recruiting	12/2025
NCT04340024 Discovery of Biomarkers for Intrinsic Radiation Sensitivity in Cancer Patients	National Cancer Centre, Singapore	Prostate (and nasopharyngeal cancers)	Observational	To identify genes or molecular pathways that are associated with radiation sensitivity in blood and tissue samples	5000	No	Recruiting	12/31/26
NCT05496959 177‐Lutetium‐PSMA Before Stereotactic Body Radiotherapy for the Treatment of Oligorecurrent Prostate Cancer, The LUNAR Study	Jonsson Comprehensive Cancer Center, Los Angeles, USA	Oligometastatic prostate cancer	Interventional	PSMA PET/CT‐based PFS for patients with oligoprogressive prostate cancer treated with SBRT to all known sites of disease on PSMA PET/CT versus patients treated with 177Lu‐PNT2002 prior to SBRT to all known sites of disease.	100	No	Recruiting	9/1/25
NCT05885009 Feasibility and Impact of Liquid Biopsy Genomic Profiling on Treatment Patients With Metastatic Prostate Cancer in Spain (SOLTI‐2102)	Barcelona, Spain	Metastatic prostate cancer	Observational	Percentage of patients for whom a liquid biopsy result can be obtained.	240	No	Recruiting	3/28/28
NCT05513638 Safety and Accuracy of Artificial Intelligence‐aided Precision MRI Assessment for the Optimization of Prostate Biopsy in Men With Suspicion of Prostate Cancer: a Multicenter Randomized Controlled Trial.	Nanjing, China	Prostate Cancer	Observational	Biopsy or surgery confirmed newly‐diagnosed prostate cancer and clinically significant prostate cancer	2000	No	Recruiting	8/22/26
NCT05697198 PRospective rEgistry OF Advanced Stage cancER (PREFER) Patients to Assess Prevalence of Actionable Biomarkers and Driver Mutations Using the OmniSeq Test and Creation of a Biobank from Community Cancer Clinics in the USA to Address Disparities in Precision Medicine	North Carolina, USA	Nine cancers including prostate cancer	Observational	The percent adoption of the OmniSeq next generation sequencing (NGS) testing platform in an advanced cancer patient population compared with baseline over a 2‐year period	2500	Yes	Recruiting	9/2024
NCT03503344 A Randomized, Phase II Study of Apalutamide +/− Stereotactic Body Radiotherapy (SBRT) in Castration‐Resistant Prostate Cancer Patients With Oligometastatic Disease on PSMA‐PET Imaging	University of California, San Francisco	Metastatic castrate‐resistant prostate cancer	Interventional	The primary endpoint for the study is the proportion of patients with undetectable serum PSA (< 0.2 ng/mL) at 6 months following completion of apalutamide therapy (18 months from date of randomization).	60	No	Recruiting	12/31/24
NCT04067570 Post‐Prostatectomy Linac‐Based Ultrahypofractionated Radiotherapy for Patients With Localized Prostate Cancer: A Treatment Feasibility and Outcomes Study	Sunnybrook Health Sciences Center, Toronto, Canada	Localized prostate cancer	Interventional	Acute genitourinary (GU) and gastrointestinal (GI) toxicities	30	No	Recruiting	10/2024
Bladder cancer								
NCT05096533 Prospective Multi‐center Clinical Study on the Application Value of Artificial Intelligence in MRI Precision Diagnosis and Treatment of Bladder Cancer	Nanjing, China	Bladder cancer	Observational	To explore the application value of artificial intelligence in the precise diagnosis and treatment of bladder tumour, and to improve the accuracy of MRI diagnosis of bladder cancer stage and grade through artificial intelligence.	150	No	Recruiting	8/2023
NCT05767528 Clinical Study of neoadjuvant FDA‐approved drugs Outcome Prediction of Muscle‐invasive Bladder Cancer Based on Patient‐derived tumour‐like cell clusters (PTC) Drug Sensitivity Detection	Bejing, China	Muscle invasive bladder cancer	Observational	Treatment response eg Complete Response (CR) or partial response (PR) – CR defined as Disappearance of all target lesions. Any pathological lymph nodes (whether target or non‐target) must have reduction in short axis to <10 mm. Length is measured in millimetres, refers to RECIST 1.1.	40	No	Recruiting	12/8/24
NCT04412070A Pilot Study to Assess the Concordance of Genomic Alterations Between Urine and Tissue to Develop Precision Medicine‐Based Immunotherapy Approaches in High‐Risk NMIBC Patients	Paris, France	High NMIBC	Observational	Agreement rate between urine cell‐free DNA and tumour tissue mutation profile	40	No	Recruiting	7/2025
NCT05415631 Augmented Bladder Tumour Detection Using the Bladder‐Portable Artefact Detection System: A Multicentric Prospective Analytic Study Using Real Time Based Artificial Intelligence (IA).	Centre Hospitalier Universitaire, Amiens, France	Bladder cancer	Observational	Tumour detection rate of white light cystoscopy and Bladder‐PAD cystoscopy	500	No	Recruiting	5/2029
NCT04410302 University of California Minority Patient‐Derived Xenograft (PDX) Development and Trial Center (UCaMP) to Reduce Cancer Health Disparities	University of California, Davis	Bladder and other solid tumours	Observational	To establish and characterize at least 200 PDXs, and utilize these PDXs in preclinicial testing of single agents and drug combinations that help guide future clinical decision‐making emphasizing the largest racial/ethnic minority populations residing in California: Hispanic/Latino Americans ([HLAs), Asian Americans/Native Hawaiians/Pacific Islanders (AANHIPIs), and African Americans (AAs) compared with non‐Hispanic Whites (NHWs).	500	Yes	Recruiting	1/2024
NCT05559177 An Open, Dose‐escalation Clinical Study of Chimeric Exosomal Tumour Vaccines for Recurrent or Metastatic Bladder Cancer	Shanghai Pudong Hospital, China	Recurrent or metastatic bladder cancer	Interventional	The percentage of patients with CR and PR in the total number of patients in the same period, as well as OS and adverse effects	9	No	Recruiting	9/1/23
NCT03693014 A Phase II Trial of Hypofractionated Radiotherapy in Patients With Limited Progression on Immune Checkpoint Blockade	Memorial Sloan Kettering Cancer Center, NY, USA	Metastatic cancers including Bladder and Renal cancer	Interventional	In non‐irradiated lesions during the first 24 weeks after treatment initiation as determined by the investigator using Response Evaluation Criteria in Solid Tumours (RECIST) 1.1 criteria in patients with limited progression receiving hypofractionated radiotherapy. ORR rate is defined as the number of patients treated to a given arm with a best overall response (BOR) of complete response (CR) or partial response (PR) divided by the total number of patients.	60	No	Recruiting	10/2023
Renal cancer								
NCT05420519 A Phase I Clinical Study of CD70‐targeted CAR‐T Therapy for Advanced/Advanced Renal Cancer	Jinan, China	Metastatic Renal Cancer	Interventional	Incidence of Adverse events after CD70 CAR‐T cells infusion, obtain the maximum tolerated dose of CD70 CAR‐T cells and Disease control rate of CAR‐T cell preparations in CD70 positive advanced malignancies	24	No	Recruiting	12/31/2024
NCT05327686 Randomized Phase II Stereotactic Ablative Radiation Therapy (SABR) for Metastatic Unresected Renal Cell Carcinoma (RCC) Receiving Immunotherapy (SAMURAI)	City of Hope Comprehensive Cancer center, Duarte, California	Advanced Renal Cancer	Interventional	Nephrectomy and radiographic progression‐free survival and percentage of participants with complete or partial response	240	No	Recruiting	6/15/28
NCT05468190 A Phase I Clinical Study to Assess the Safety and Tolerability of CD70‐targeting CAR‐T in the Treatment of CD70‐positive Advanced/Metastatic Solid Tumours	Zhengzhou, Henan, China	Metastatic solid tumours including renal cell carcinoma	Interventional	Incidence of Adverse events after CD70 CAR‐T cells infusion, obtain the maximum tolerated dose of CD70 CAR‐T cells and Disease control rate of CAR‐T cell preparations in CD70 positive advanced malignancies	48	No	Recruiting	7/17/25
NCT03592472 A Randomized, Phase 3, Double‐blind, Placebo‐controlled Study of Pazopanib With or Without Abexinostat in Patients With Locally Advanced or Metastatic Renal Cell Carcinoma (RENAVIV)	Dover, Delaware	Renal Cell Carcinoma	Interventional	To compare the PFS between treatment arms	413	No	Recruiting	6/30/23
NCT05703269 Hypofractionated Radiotherapy vs Single Fraction Radiosurgery for Brain Metastasis Patients on Immunotherapy (HYPOGRYPHE)	Bronx, New York, USA	Brain metastases patients including those with primary renal cell carcinoma	Interventional	Occurrence of a Grade 2 or higher Adverse Radiation Effect and comparison of time to local failure and mortality	244	No	Recruiting	3/2028
NCT05830058 Phase II Randomized Controlled Trial of Biologically Guided Stereotactic Body Radiation Therapy in Oligoprogressive Non‐Small Cell Lung Cancer, Melanoma, and Renal Cell Carcinoma	City of Hope Comprehensive Cancer center, Duarte, California	Non small cell lung, Melanoma and renal cell carcinoma	Interventional	Feasibility and safety of positron emission tomography (PET) adaptive stereotactic body radiation therapy (SBRT), time to progression, overall survival and quality of life	32	No	Recruiting	6/1/26
Multiple cancer trials including GU cancers								
NCT05199584 A Phase 2, Multi‐Center Study Evaluating the Safety and Efficacy of ENV‐101 (Taladegib) in Patients With Advanced Solid Tumours Harbouring PTCH1 Loss of Function Mutations	Endeavour Clinical Trials, San Antonio, Texas	Solid tumours including GU Cancers	Interventional	ORR is comprised of Complete Response (CR) and Partial Response (PR), measured by Response Evaluation Criteria in Solid Tumours, Version 1.1 (RECIST 1.1), as determined by an independent review (confirmed CR or PR will be defined as a repeat assessment performed no less than 28 days after the criteria for response is first met).	44	No	Recruiting	5/2024
NCT03896958 The PIONEER Initiative: Precision Insights On N‐of‐1 Ex Vivo Effectiveness Research Based on Individual Tumour Ownership (Precision Oncology)	Georgia and Washington, USA	All cancers including all GU Cancers	Observational	The primary mission of our team is to assess benefit of patient tissue ownership and functional testing to the standard of care process. We will calculate this as a % of patients.	1000	No	Recruiting	3/12/24
NCT03452774 SYNERGY‐AI: Artificial Intelligence Based Precision Oncology Clinical Trial Matching and Registry	New York, USA	All cancers including all GU Cancers	Observational	International registry for cancer patients evaluating the feasibility and clinical utility of an Artificial Intelligence‐based precision oncology clinical trial matching tool and its clinical impact on pts with advanced cancer to facilitate clinical trial enrollment (CTE).	50 000	Although not a specific race diverse initiative, the trial will aim to identify barriers to accruals to clinical trials, as measured and reported by a questionnaire	Recruiting	6/2025
NCT05786716 DETERMINE Trial Treatment Arm 4: Trastuzumab in Combination With Pertuzumab in Adult, Teenage/Young Adult and Paediatric Patients With Cancers With HER2 Amplification or Activating Mutations	Cancer Research UK, Manchester, UK	Solid cancers including all GU cancers	Interventional	Objective disease response and durable clinical benefit	30	No	Recruiting	10/2029
NCT04354064 Circulating Tumour DNA (ctDNA) for Early Treatment Response Assessment of Solid Tumours	Washington University School of Medicine, St Louis USA	Solid tumours including GU cancers	Observational	Freedom from progression ‐Defined as RECIST 1.1 based radiographic or clinical progression, with non‐progressors censored at last radiographic follow‐up	3362	No	Recruiting	12/31/25

An early definition of PM took into consideration various patient characteristics, including age, family history and performance status, but has now expanded to include molecular, pathological, lifestyle or genomic factors when available.^37^ The future vision for PM aims to include integrated ‘omics’ data which may reveal insights into tumour biology through study not only of genomics but also transcriptomics, proteomics, metabolomics, immune pathways and the microbiome. Our increasing understanding of these cellular pathways and their mechanistic links to the pathogenesis of cancer has led to the creation of many targeted systemic agents for a variety of cancers, including GU cancers.^38^, ^39^

### 3.1 Prostate cancer

Molecular tumour profiling and the identification of targetable mutations are a prime focus for PM PCa research, and there are, for example, several androgen receptor pathway inhibitors available that have traditionally been mainstays of treatment.

#### 3.1.1 PTEN‐Pl3K‐AKT pathway inhibitors

For example, increasingly these medications are employed earlier in the disease course making castrate resistance more likely to develop,^40^, ^41^ and mutations in the PTEN‐PI3K‐AKT pathway are often seen in mCRPCa.^42^ In an analysis of castrate‐resistant patients treated with abiraterone after docetaxel, patients with PTEN mutations experienced worse overall survival (OS). Further studies, including the randomized phase III IPATential150 trial, may more clearly define the role of AKT inhibitors but, as the authors have found is a common problem in PM research, the protocol does not seem to include active recruitment for racially diverse patients.^43^, ^44^ Table 2 summarizes the trials found in the literature focused on mCRPC with cohorts including AA men.

**TABLE 2:** Clinical trials in Black men with metastatic prostate cancer.

Reference	Study design	Cohort size	Drug(s)	Endpoint	Result
George et al., 2023	Phase 2 non randomized prospective comparative trial	93 men (43 [44%] Black men) with mCRPC	Combination apalutamide and abiraterone with prednisone	Radiographic progression free survival (rPFS), overall mortality (OM) and PSA response	Better rPFS in Black men (30 months) compared with White men (15 months). OM at 2 years 14% for Black men compared with 33% in White men. 93% of Black men had a >50% decline compared with 68% of White men
Shore et al., 2022	Subanalysis of the phase 3 randomized controlled ARAMIS trial	1509 men (52 [3.4%] Black men) with non metastatic CRPC	Darolutamide	Metastasis free survival (MFS) and overall survival	Black men demonstrated improved MFS and OS consistent with the overall ARAMIS trial cohort
Zhao et al., 2020	Retrospective analysis	318 men (87 [27%] Black men) with mCRPC	Radium 223	Overall mortality	Compared with White men, Black men had a 25% lower risk of mortality from the time of starting radium 223 (HR 0.75, CI 0.57–0.99; *p* < 0.045)
Sartor et al., 2020	Retrospective analysis of a cohort from PROCEED FDA‐requested registry	1902 men (221 [12%] Black men) with mCRPC	Sipuleucel‐T	Overall survival (OS)	Compared with White men, Black men had improved OS especially in the group with lower baselines PSAs (54.3 months vs. 33.4 months) (HR 0.52, CI 0.37–0.72; *p* < 0.001).
Halabi et al., 2019	Metanalysis of nine phase 3 Trials	8820 men (500 [6%] Black men) with mCRPC	Docetaxel	Overall survival (OS)	Adjusting for known negative prognostic indicators, multivariable analysis showed a significantly reduced risk of death for Black men compared with White men (HR 0.81, CI 0.72–0.91; *p* < 0.001)
McNamara et al., 2019	Retrospective analysis	2910 men (787 [27%] Black men) with mCRPC	Abiraterone or Enzalutamide	Overall survival (OS)	Compared with White men, Black men had better OS (918 days vs. 781 days) (HR 0.826, CI 0.732–0.933; *p* < 0.002)
Ramalingham et al., 2017	Prospective case control study	135 men (45 [33%] Black men) with mCRPC	Abiraterone	PSA response	Compared with White men, a higher proportion of Black men had >50% and >30% PSA responses at 68.9% versus 48.9% (*p* < 0.028)) and 77.8% versus 54.4% (*p* < 0.008), respectively.
Efstathiou et al., 2014	Randomized double blind phase 3 trial	1088 men (28 (2.6%) Black men with mCRPC	Abiraterone	PSA response and time to PSA progression (TPP)	Compared with White men, a higher proportion of Black men had >90% PSA response and TPP with 53.3% versus 31% and 16.6 months versus 11.1 months, respectively

#### 3.1.2 Antiandrogen agents

Trials using the novel oral antiandrogen abiraterone in a prospective cohort of prechemotherapy mCRPCa patients demonstrated an enhanced PSA response in Black men (greater than 90% drop in PSA in over 50% of patients compared with only 30% of White patients),^45^ as did a similar study with equitable access for Black men with nearly 70% of Black men halving their PSA compared with 49% of White men.^46^ Survival rates for Black men with mCRPCa are similarly more favourable than for White men treated with novel oral antiandrogens, with studies showing 918 versus 781 days OS, respectively, using either enzalutamide or abiraterone^47^ and 16.6 versus 11.5 months PSA progression free survival, respectively, with abiraterone.^48^ More recently, early results from the PANTHER trial, suggest Black men with mCRPCa also have a better response to combined abiraterone and apalutamide [an AR receptor inhibitor] with prednisolone than White men.^49^ The efficacy of these treatments in other racial groups requires further study.

#### 3.1.3 Autologous cellular immunotherapy

In general, immunotherapies have been less successful in PCa with the notable exception of the Sipuleucel‐T vaccine. Data from several studies examining patients with advanced PCa found enhanced OS with the addition of Sipuleucel‐T.^50^, ^51^, ^52^ Moreover, some data suggest that AA patients with PCa may respond better to Sipuleucel‐T than CAs. A registry study of PSA‐matched patients receiving Sipuleucel‐T found an OS of 35.3 months for AAs versus 25.8 months for CAs.^53^

#### 3.1.4 Checkpoint inhibitors

These results are consistent with other studies that suggest that AAs with PCa exhibit a more pronounced upregulation of immune signalling pathways.^54^ As discussed above, several genes involved in inflammatory pathways and immune checkpoint inhibition (ICI), such as IFN‐G, CCL4 and IL33, and immune biomarkers, including CD3, ICOSLG and PDL2, are expressed at significantly higher levels in tumours from AAs,^14^ which may underlie their improved efficacy with carefully chosen chemo‐ and immuno‐therapy regimes. A large metanalysis also found improved survival in Black men with mCRPCa with Docetaxel on multivariable analysis pooling 9 phase 3 trials with nearly 9000 men, 500 of whom were Black.^55^

#### 3.1.5 Abscopal effect

Lastly, there has been increased interest in combining immunotherapies with radiation therapy (RT) to utilize ‘the abscopal effect’ resulting from RT‐induced immune activation and upregulation of inflammatory chemokines resulting in tissue‐ and tumour‐infiltration by antigen‐presenting and cytotoxic lymphocytes to reduce disease burden at distant sites.^56^, ^57^ Immunotherapy may then leverage the immune system via a myriad of mechanisms, including anti‐tumour vaccination and ICI to prevent the apoptosis of cytotoxic T‐cells.^58^, ^59^ Intuitively, racial differences in tumour biology resulting in enhanced immune signalling discussed above may have synergy with this abscopal effect and combination therapy with other treatment modalities, such as radiotherapy, which has been most extensively studied, may thus yield better results.^60^, ^61^, ^62^

In clinical settings, stereotactic body radiation therapy (SBRT) has been used to trigger antitumour immunity in patients on immunotherapy based on the rationale that combining these treatment modalities may increase their collective efficacy as they both reduce regulatory T cells and increase tumour infiltration by lymphocytes.^63^ A recent study combined SBRT and immunotherapy in men with mCRPC with at least one bone metastasis who had progressed after receiving docetaxel. The subjects were randomized in a 1:1 ratio to receive bone‐directed RT followed by either ipilimumab or placebo. While the primary endpoint of OS was not improved, reductions in PSA and an improvement in progression‐free survival were noted.^64^ The use of radiopharmaceuticals in combination with targeted immunotherapy has also been studied, and a retrospective analysis of over 300 men with mCRPCa receiving radium‐223 confirmed improved survival for Black men.^65^ Nevertheless, further studies exploring the effects of combining immunotherapy and SBRT across different racial groups are urgently needed.

### 3.2 Bladder cancer

As mentioned above, PM BCa initiatives have targeted actionable mutations in several genes, including TP53, PIK3CA, CDKN1A, ERBB 2 and FGFR.^66^ This has led to therapeutic tools with promise, but in reality, there is a paucity of study across different racial groups.

#### 3.2.1 FGFR targeting agents

For example, FGFR has been the focus of several studies with numerous putative targeted agents^67^ and although the results of a phase II trial with 90 patients showed response rates of nearly 70% to an agent targeting FGFR2 and FGFR3, the ethnicity of patients in the cohort was not published.^68^ Additionally, a study comparing the efficacy of erdafitinib versus chemotherapy or pembrolizumab in patients with advanced BCa harbouring selected FGFR aberrations who have progressed after 1 or 2 prior therapies is currently accruing patients, but the protocol does not include an initiative for racially diverse recruitment.^69^

#### 3.2.2 Checkpoint inhibitors

Similarly in KEYNOTE‐052, first‐line pembrolizumab in cisplatin‐ineligible patients with locally advanced or metastatic BCa can lead to a durable clinical response and a prolonged OS.^70^ More recently, maintenance therapy with Avelumab has been shown to lead to a statistically significant improvement in OS over best supportive care in patients with unresectable or metastatic BCa who did not progress after first‐line chemotherapy.^71^ Neither of these important studies published racial demographics in their findings further supporting the sentiment that racially diverse PM research to offset racial inequities is underrepresented.

### 3.3 Renal cancer

The situation is somewhat different for RCC, where research has yielded useful insights for AA men, although NA and HA populations remain underrepresented.

#### 3.3.1 Checkpoint inhibitors

In addition to the tyrosine kinase inhibitors targeting VEGF pathways, studies have shown that a combination of targeted therapeutics plus immunotherapy agents can nearly double the survival of patients with RCC.^72^ These findings were confirmed when Checkmate 025 and 214 helped cement the role of ICIs in the management of RCC.^73^, ^74^ This is a particularly important finding in the treatment of AA patients, because, as has been discussed, they are less likely to have VHL mutations thus rendering agents that act on the VEGF pathway less effective.

#### 3.3.2 Abscopal effect

Knowing that certain systemic treatments may not be as effective in AA patients makes exploring the possible role of SBRT more significant. Current studies investigating the role of SBRT in RCC suggest that exploiting the abscopal effect in combination with immunotherapy may improve efficacy for oligometastatic disease in combination with VEGF targeting agents.^75^ Once again, more study is required.

## 4 CONCLUSION

In the realm of GU cancers, both SDOH and cytogenetic factors play a pivotal role in determining patient outcomes. Unfortunately, AA, HA and NA patients have suffered from a lack of access to quality healthcare, in part due to socioeconomic factors such as lower rates of health insurance, racial biases and geography.^76^ Adding to this issue is their historical underrepresentation in clinical trials, despite the Food and Drug Administration's attempts to diversify enrollment.^77^ Nevertheless, as our understanding of differences in how social, biological and genetic factors influence treatment responses in racially diverse groups improves, clinicians have increasingly been able to direct novel treatments to these populations in an efficacious manner, narrowing the gap in outcomes between AA patients and other races.

As PM becomes increasingly incorporated into treatment planning in the future, underrepresented populations must also be included in all research efforts. PM can be a valuable tool in narrowing the gap in outcomes across ethnic groups. Although further studies are needed, by promoting minority enrollment in trials we will be able to further substantiate the role of PM in this setting.

## AUTHOR CONTRIBUTIONS

Kunal S. Sindhu wrote the manuscript with contributions from Zachary Dovey, Marcher Thompson, Anthony D. Nehlsen, Karin A. Skalina, Beata Malachowska, Shaakir Hasan, Chandan Guha, Justin Tang and Lucas Resende Salgado.

## CONFLICT OF INTEREST STATEMENT

Dr. Zach Dovey is Medical Director and stock owner (with certificate of shares) of Medtech Holdings Ltd. No other authors have any conflicts to declare.
